# Comparative analysis of absent in melanoma 2-inflammasome activation in *Francisella tularensis* and *Francisella novicida*

**DOI:** 10.3389/fmicb.2023.1188112

**Published:** 2023-05-17

**Authors:** Maha Alqahtani, Zhuo Ma, Jacob Miller, Jen Yu, Meenakshi Malik, Chandra Shekhar Bakshi

**Affiliations:** ^1^Department of Pathology, Microbiology and Immunology, New York Medical College, Valhalla, NY, United States; ^2^Department of Basic and Clinical Sciences, Albany College of Pharmacy and Health Sciences, Albany, NY, United States; ^3^Electron Microscopy Laboratory, Department of Pathology, Westchester Medical Center, Valhalla, NY, United States

**Keywords:** *Francisella tularensis*, *novicida*, inflammasome, Aim2, immune suppression

## Abstract

*Francisella tularensis* is a highly virulent Gram-negative bacterium that causes the fatal zoonotic disease tularemia. The mechanisms and signaling pathways leading to the absent in melanoma 2 (Aim2) inflammasome activation have been elegantly elucidated using *Francisella novicida* as a model. Although not pathogenic for humans, *F*. *novicida* can cause tularemia in mice, and the inflammatory response it triggers is the polar opposite to that observed in mice infected with *F*. *tularensis* strains. This study aimed to understand the mechanisms of Aim2 inflammasome activation in *F*. *tularensis-*infected macrophages. The results reveal that macrophages infected with the *F*. *tularensis* live vaccine strain (LVS) induce lower levels of Aim2-dependent IL-1β than those infected with *F*. *novicida*. The suppression/weak activation of Aim2 in *F*. *tularensis* LVS-infected macrophages is due to the suppression of the cGAS-STING DNA-sensing pathway. Furthermore, the introduction of exogenous *F*. *tularensis* LVS DNA into the cytosol of the *F*. *tularensis* LVS-infected macrophages, alone or in conjunction with a priming signal, failed to restore IL-1β levels similar to those observed for *F*. *novicida-*infected macrophages. These results indicated that, in addition to the bacterial DNA, DNA from some other sources, specifically from the damaged mitochondria, might contribute to the robust Aim2-dependent IL-1β levels observed in *F*. *novicida*-infected macrophages. The results indicate that *F*. *tularensis* LVS induces mitophagy that may potentially prevent the leakage of mitochondrial DNA and the subsequent activation of the Aim2 inflammasome. Collectively, this study demonstrates that the mechanisms of Aim2 inflammasome activation established for *F*. *novicida* are not operative in *F*. *tularensis*.

## Introduction

*Francisella tularensis* (*F*. *tularensis*) is a non-motile, facultative intracellular, Gram-negative bacterium that causes a fatal zoonotic disease, tularemia. *Francisella tularensis* is classified into *F*. *tularensis* subspecies *tularensis, F*. *tularensis* subspecies *holarctica,* and *F*. *tularensis* subspecies *mediasiatica*. *Francisella novicida* is classified as a separate species because it is genetically and phenotypically different from *F*. *tularensis* (Kingry and Petersen, 2014). While *F*. *tularensis* subspecies *tularensis* and *F*. *tularensis* subspecies *holarctica* cause tularemia in humans, *F*. *novicida* is associated with disease in immunocompromised individuals only (Pechous et al., 2009; Sjödin et al., 2012). *Francisella tularensis* subspecies *tularensis* (also known as Type A strains) has a very high virulence in humans, with a lethal dose that could be lower than 10 colony colony-forming (CFUs) and is responsible for the vast majority of tularemia cases in North America. Due to its extremely high virulence, high rate of morbidity and mortality, and ease of aerosolization, *F*. *tularensis* subspecies *tularensis* SchuS4 strain is classified by the CDC as a Tier 1 Category A Select Agent (Ellis et al., 2002). *Francisella tularensis* subspecies *holarctica* (Type B strains) cause tularemia in healthy individuals. However, they are less virulent compared to *F*. *tularensis* subspecies *tularensis* strains, with a lethal dose of 10,000 CFUs or less (Pechous et al., 2009). The live vaccine strain (LVS) was developed by Russian scientists through serial *in vivo* and *in vitro* passages of *F*. *tularensis* subspecies *holarctica,* resulting in attenuation of its virulence in humans. The *F*. *tularensis* LVS was later gifted to the United States by the former Soviet Union in the 1950s (Eigelsbach and Downs, 1961). *Francisella tularensis* LVS shares a high DNA sequence homology and undergoes an identical intramacrophage replication cycle with highly virulent *F*. *tularensis* strains such as SchuS4. Hence, in research laboratories, *F*. *tularensis* LVS is used to study the virulence factors of *Francisella* and the pathogenesis of tularemia (Jones et al., 2014).

*Francisella tularensis* is transmitted to humans or animals by contact with infected materials, consumption of contaminated food and water, inhalation of aerosols caused by the disruption of materials containing the organism, and through bites of infected ticks, mosquitoes, and flies (Dennis et al., 2001). Depending on the route of bacterial entry, the clinical manifestations range from ulceroglandular, oculoglandular, and respiratory forms of tularemia (Dennis et al., 2001). The extreme virulence of *F*. *tularensis* ultimately depends on its ability to enter, persist, and replicate within the host cells without evoking an immediate innate immune response. *Francisella* enters macrophages through phagocytosis by forming asymmetric pseudopod loops (Clemens et al., 2005). Following internalization, bacteria initially reside in the phagosome and prevent its fusion with lysosomes (Golovliov et al., 2003; Clemens et al., 2004; Santic et al., 2005). Within 30 min to several hours, phagosomal membrane disruption and bacterial escape to the cytosol occur. Once in the cytosol, *Francisella* undergoes several rounds of replication that end with the death of the host cell (Lai et al., 2001; Oyston et al., 2004; Mariathasan et al., 2005; Santic et al., 2010).

In cytosol, the sensing of foreign nucleic acids is a major anti-microbial immune mechanism. The nucleic acid sensors include the retinoic acid-inducible gene (RIG)-I-like receptors (RLRs), NOD-like receptors (NLRs), and absent in melanoma 2-like receptors (ALRs). Additionally, DNA sensors that induce the production of Type I interferons (Type-I IFNs) include cyclic-GMP-AMP synthase (cGAS), the stimulator of interferon genes (STING), DNA-dependent activator of IFN-regulatory factors (DAI), and leucine-rich repeat flightless-interacting protein 1 (LRRFIP1; Takaoka et al., 2007; Burdette et al., 2011). All these sensors recognize pathogen-associated molecular patterns (PAMPs) and/or sterile endogenous molecules produced by the host cell tissue injury that are called damage-associated molecular patterns (DAMPs). Recognition of PAMPs or DAMPs initiates several signaling pathways, leading to the induction of proinflammatory cytokines and interferons via MyD88-dependent NF-κB and interferon-regulatory factors (IRFs) pathways, respectively (Kaiser et al., 2008). Absent in Melanoma 2 (AIM2) is an interferon inducible ALR family protein. AIM2 contains a C-terminal HIN-200 domain, which binds directly to cytosolic double-stranded DNA and an N-terminal pyrin motif, which recruits and interacts with ASC (Fernandes-Alnemri et al., 2009). AIM2 detects bacterial and viral DNA and senses mislocalized self-DNA released into the cytosol during inflammatory and autoimmune diseases (Hornung et al., 2009).

The majority of the studies related to cytosolic recognition of *Francisella* and activation of the AIM2 inflammasome have been done using *F*. *novicida*. Once the host cell is infected with *F*. *novicida,* a robust AIM2-dependent inflammasome response is induced, culminating in the production of a potent proinflammatory cytokine response and pyroptosis to clear the infection. In agreement with cell-based assays, Aim2, Caspase-1, and Asc are required for survival during *F*. *novicida* infection in mice (Mariathasan et al., 2005; Fernandes-Alnemri et al., 2010; Jones et al., 2010; Rathinam et al., 2010). Mice lacking either caspase-1 or Asc have a higher bacterial burden in the lung, liver, and spleen after 2 days, and 100% of them die within 3–5 days post-infection. In contrast, 25–30% of wild-type mice survive the infection(Mariathasan et al., 2005; Fernandes-Alnemri et al., 2010; Jones et al., 2010). Additionally, several studies using *F*. *novicida* have shown the critical protective role of inflammasome-induced IL-1β cytokine (Mariathasan et al., 2005; Li et al., 2006; Henry and Monack, 2007).

It has been shown that there is a delay in the activation of the inflammasome and significantly lower levels of IL-1β are produced by macrophages infected with *F*. *tularensis* LVS, as compared to those infected with *F*. *novicida* (Dotson et al., 2013). Similar to *F*. *tularensis* LVS, *F*. *tularensis* SchuS4-infected macrophages also fail to induce early inflammasome activation and IL-1β production. On the other hand, *F*. *novicida-*infected macrophages strongly activate the inflammasome. It has been established that infection of murine macrophages with *F*. *novicida* leads to the induction of IFN-β in a cGAS-STING-dependent manner, which subsequently binds to interferon-alpha/beta receptor in an autocrine fashion. This binding leads to the induction of multiple transcription factors that drive the expression of interferon-stimulated genes (ISGs) and guanylate binding proteins (GBPs), causing bacterial cell lysis by damaging the bacterial cell wall and the release of bacterial DNA in the host cell cytosol (Henry et al., 2007; Man et al., 2015; Storek et al., 2015). The released DNA is sensed by AIM2, which leads to the recruitment of the adaptor ASC and the cleavage of pro-caspase1 into its active form. The active caspase1 acts as a biological scissor and cleaves immature pro-IL-1β and pro-IL-18 into their active forms. Caspase1 also cleaves gasdermin D (GSDMD) into N and C terminal domains. The cleaved N-terminal domain of GSDMD forms pores in the cell membrane, that allows the secretion of bioactive forms of IL-1β and IL-18. The potassium efflux caused by the perforation of cell membrane eventually leads to the lytic form of cell death known as pyroptosis (Shi et al., 2015). Thus, the mechanisms and signaling pathways involved in AIM2 activation are known for the human avirulent *F*. *novicida* strain. However, these mechanisms remain unknown for the human virulent *F*. *tularensis* strains. This study aimed to investigate the innate immune evasion mechanisms of *F*. *tularensis* LVS, specifically those that lead to suppression/weak activation of AIM2 inflammasome and contribute to restricting early inflammation, and how these mechanisms differ between *F*. *tularensis* LVS and *F*. *novicida*.

## Materials and methods

### Animals

Six to eight-week-old mice were used in experiments. *Aim2*^−/−^ [B6. 129P2-*Aim2^Gt(CSG445)Byg^/J;* Stock number:013144, Jackson Laboratory] and the corresponding wild-type C57BL/6 mice (000664 C57BL/6 J, Jackson Laboratory) were housed in a pathogen-free environment in the Animal Resource Facility of New York Medical College. All experiments were conducted according to the protocols approved by the institutional animal care and use committee (IACUC).

### Bacterial strains

All work with *F*. *tularensis* subspecies *holarctica* LVS and *F*. *novicida* (BEI Resources, Manassas, VA, United States) was carried out in a Bio-Safety Level-2 laboratory. Bacterial stocks were prepared by growing bacteria on Mueller-Hinton (MH) agar plates at 37°C with 5% CO_2_. The individual colonies were picked from MH-agar plates and grown overnight in MH-broth (containing 10% glucose, 0.021% anhydrous calcium chloride, 0.000138% hydrous magnesium chloride, 2.5% ferric pyrophosphate, and 2.5% Isovitalex). Aliquots of mid-log phase bacteria (OD_600_ of 0.2) were snap-frozen and stored at −80°C. A vial of the frozen stock was thawed at 37°C and then plated on MH-agar and grown at 37°C with 5% CO_2_ for 48 h for use in the experiments.

### Bone marrow-derived macrophages and cell culture assays

Bone marrow cells isolated from 6- to 8-week-old wild-type and *Aim2^−/−^* knockout mice were differentiated into bone marrow-derived macrophages (BMDMs) as previously described (Toda et al., 2021). Briefly, bone marrow cells were cultured in Dulbecco’s modified Eagle medium (DMEM) containing 10% heat-inactivated fetal bovine serum (FBS), 2% l-glutamine, 1% sodium pyruvate, 1% HEPES, and 20% medium conditioned with L929 cells. The differentiated BMDMs were plated in 12- or 24-well plates and incubated overnight at 37°C in the presence of 5% CO_2_ prior to use in experiments. Immortalized Gasdermin D (*Gsdmd^−/−^*) BMDMs and their corresponding wild-type immortalized BMDMs were kindly provided by Dr. Vijay Rathinam, UCONN Health. BMDMs from *Sting^−/−^* mice were kindly provided by Dr. Penghua Wang, UCONN Health.

The BMDMs were infected with *Francisella* cultures at a multiplicity of infection (MOI) of 100:1 (Bacteria: cell ratio). Infection was synchronized by centrifuging the plate at 1,000 rpm at 4°C for 10 min, followed by incubation for 2 h at 37°C with 5% CO_2_. After 2 h, the medium was replaced with medium containing 250 μg/mL of gentamicin to kill all the extracellular and adherent bacteria. The plate was incubated again for 1 h at 37°C in the presence of 5% CO_2_. After 1 h of incubation, the medium was replaced with medium without any antibiotic. Culture supernatants were collected at 6-, 12-, and 24-h post-infection and stored at −80°C for cytokines analysis. Cells were lysed at 4- and 24-h post-infection or as otherwise indicated, with 0.1% sodium deoxycholate, diluted 10-fold serially, plated on MH-chocolate agar plates, and incubated at 37°C in the presence of 5% CO_2_ for 48 h. The colonies were counted, and the results were expressed as Log_10_ colony forming units (CFU)/mL.

### Cell death assays

Bone marrow-derived macrophages were seeded in a 24-well plate and incubated at 37°C in the presence of 5% CO_2_ overnight. The following day, the BMDMs were infected with bacteria at an MOI of 100. Cell death was measured by lactate dehydrogenase (LDH) release in the cell culture supernatants using the CyQUANT LDH Cytotoxicity Assay Kit (Invitrogen) according to the manufacturer’s instructions.

### Isolation of *Francisella tularensis* genomic DNA

The genomic DNA from *F*. *tularensis* LVS was purified using the Genomic DNA Mini Kit (Invitrogen) according to the manufacturer’s instructions. DNA concentration and purity were measured with a spectrophotometer and stored at −20°C for future use.

### DNA transfection

Wild-type BMDMs (1 × 10^6^ cells/well) were infected with *F*. *tularensis* LVS or *F*. *novicida* for 2 h and then transfected with 1, 4, and 8 μg of *F*. *tularensis* LVS DNA using DOTAP liposomal transfection reagent. One microgram of *F*. *tularensis* LVS DNA is approximately equivalent to 1.6 million copies of the *F*. *tularensis* LVS genome. BMDMs infected with *F*. *tularensis* LVS but not transfected with DOTAP: DNA and uninfected BMDMs were used as controls. Cell culture supernatants were collected at various time intervals post-infection to determine levels of IL-1β, IFN-β, and TNF-α by ELISA.

### Cytokines analysis

Cell culture supernatants were collected from the infected and/or DNA-transfected BMDMs after 6, 12, and 24 h. Interferon-beta (IFN-β), IL-1β, and TNF-α levels were measured using LEGEND MAX™ Mouse ELISA Kits (Biolegend) according to the manufacturer’s instructions. The results were expressed as pg./mL.

### Western blot analysis

Bone marrow-derived macrophages were infected with *F*. *tularensis* LVS or *F*. *novicida* for 6 h and then lysed using RIPA lysis buffer containing a protease and phosphatase inhibitor cocktail. The protein concentrations were determined by the Pierce BCA Protein Assay Kit (Thermo Fisher Scientific, United States). An equal amount of protein from each sample were loaded onto a 10% Sodium dodecyl-sulfate polyacrylamide gel, and separated by electrophoresis. The proteins were then transferred to a 0.22 μM polyvinylidene difluoride (PVDF) membrane. The membrane was blocked for 1 h in 5% nonfat milk. The membrane was incubated overnight at 4°C with primary antibodies diluted in 5% Bovine Serum Albumin (BSA) against PINK1 (Novus Biologicals, United States), LC3A/B, and β-actin (Cell Signaling Technology, United States). The following day, the membrane was washed and incubated with 1:20,000 HRP-conjugated secondary antibodies at room temperature for 1 h. The membrane was washed and then incubated with Pierce ECL Plus Substrate. The protein bands were viewed using an X-ray Imager. The band intensities on the blots were quantified using the Bio-Rad Image Lab™ Software Version 3.0.

### Transmission electron microscopy

The BMDMs were infected with either *F*. *tularensis* LVS or *F*. *novicida* at an MOI of 100, as described previously. The uninfected BMDMs were used as controls. At 6- and 12-h post-infection, the cells were prepared for TEM as described earlier (Steele et al., 2019) and then examined under the transmission electron microscope (Hitachi HT7700). The identification of *F*. *tularensis* LVS and *F*. *novicida* was made based on the size, shape, and location of the bacteria inside the macrophages. Detection of autophagic vacuoles characterized by the presence of double-membrane vacuoles; the extent of mitophagy characterized by engulfment of mitochondria by autophagic vacuoles, and mitochondrial dynamics defined by the presence of elongated and fragmented mitochondria was performed. Quantification of the number of autophagic vacuoles, engulfed mitochondria, and elongated and fragmented mitochondria was performed by counting these structures in 10 micrographs per group.

### Statistical analysis

All data were statistically analyzed by one-way ANOVA followed by Tukey–Kramer multiple comparison test. The data were presented as Mean ± Standard error of the mean (SEM), and the differences between the experimental groups were considered statistically significant at a *p* < 0.05 level.

## Results

### *Francisella tularensis* LVS-infected macrophages produce low levels of IL-1β

**Figure 1:**
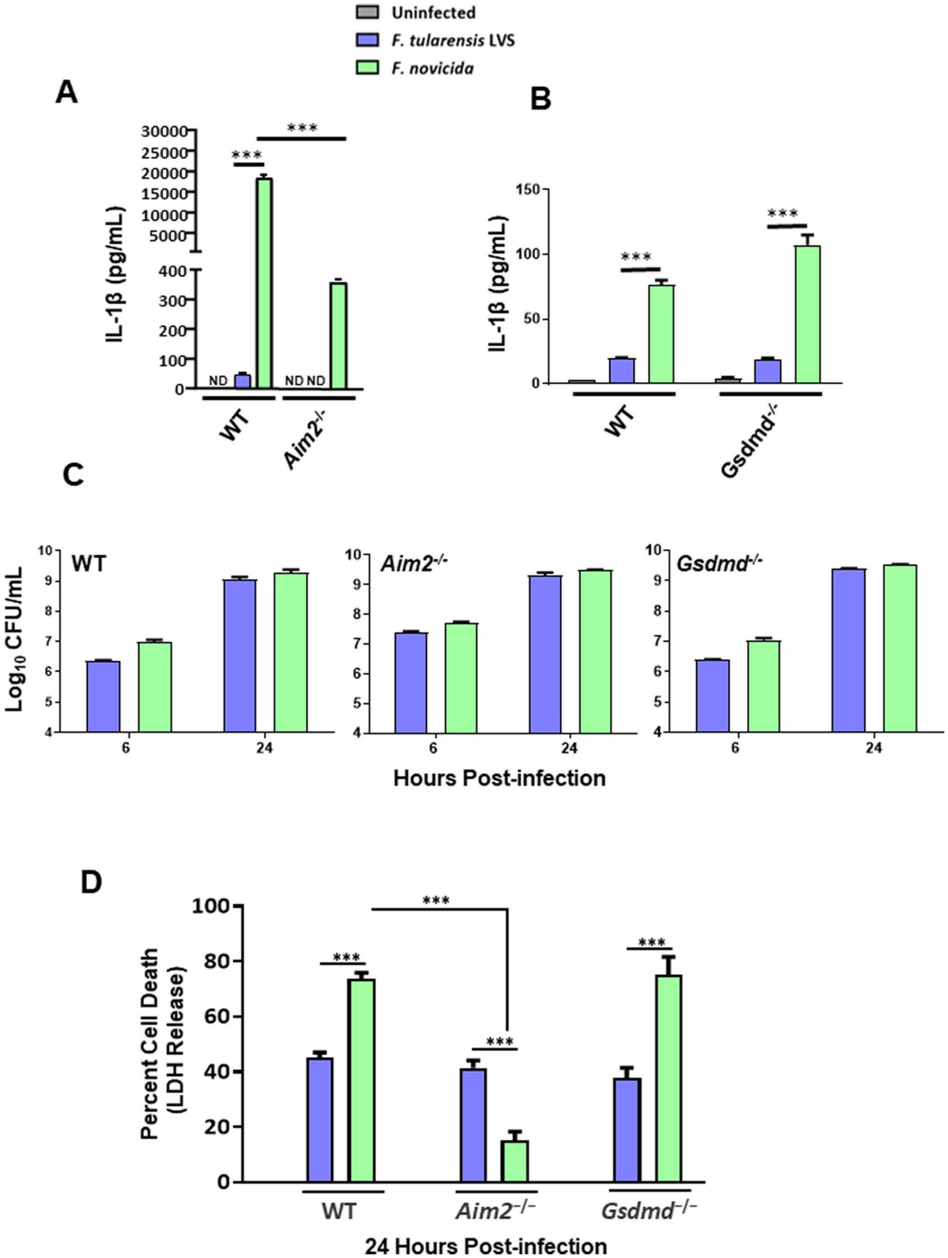
*Francisella tularensis* live vaccine strain (LVS) infection in macrophages leads to low levels of IL-1β production. Wild-type (WT), and *Aim2^−/−^* bone marrow-derived macrophages (BMDMs) **(A)** and immortalized WT and *Gsdmd^−/−^* BMDMs **(B)** were infected with *F*. *tularensis* LVS or *Francisella novicida* (MOI 100). ELISA was used to analyze the secreted levels of IL-1β in the culture supernatants 24 h post-infection. Results are expressed as pg./mL (mean ± SEM) and are cumulative of three independent experiments with up to six biological replicates. The *p* values were determined using one-way ANOVA. ^***^p < 0.001. **(C)** The same cell lines were infected with *F*. *tularensis* LVS or *F*. *novicida* (MOI 100) for 2 h, followed by gentamycin treatment for an hour to kill extracellular bacteria. Intracellular bacteria were enumerated at 6 and 24 h post-infection. Results are expressed as Log_10_ CFU/mL (mean ± SEM) and are cumulative from two independent experiments, each with three biological replicates. **(D)** The cell culture supernatants collected at 24 h from WT and *Aim2^−/−^* BMDMs and immortalized *Gsdmd^−/-^*BMDMs infected with *F*. *tularensis* LVS, or *F*. *novicida* (MOI 100) were analyzed for LDH release. Results are expressed as a percentage of LDH release and are cumulative from three independent experiments with up to six biological replicates. The *p*-values were determined using one-way ANOVA. ^***^p < 0.001. ND, not detected.

Bone marrow-derived macrophages from wild-type and *Aim2^−/−^* mice and immortalized *Gsdmd^−/−^* BMDMs and their wild-type counterparts were infected with 100 MOI of *F*. *tularensis* LVS and *F*. *novicida*. Uninfected macrophages served as negative controls. The culture supernatants from the infected and control BMDMs were analyzed 24 h post-infection for secreted levels of IL-1β. *F*. *tularensis* LVS-infected wild-type BMDMs induced significantly low levels of IL-1β as compared to *F*. *novicida*-infected BMDMs. No detectable levels of IL-1β were observed in the culture supernatants of *F*. *tularensis* LVS*-*infected *Aim2^−/−^* BMDMs, while in those from *F*. *novicida-*infected *Aim2^−/−^* BMDMs, these levels were significantly lower than those observed for the wild-type BMDMs (Figure 1A). A trend similar to that observed for the wild-type and *Aim2*^−/−^ BMDMs was also observed for the immortalized wild-type and *Gsdmd^−/−^* macrophages. However, the magnitude of IL-1β response was much lower than that observed for the primary BMDMs (Figure 1B). Overall, these results demonstrate that *F*. *tularensis* LVS-infected primary or immortalized BMDMs produce very low levels of Aim2-dependent IL-1β. On the other hand, very high levels of IL-1β observed in *F*. *novicida-*infected BMDMs were dependent on Aim2 and GsdmD.

### Differences in IL-1β levels in *Francisella tularensis* LVS and *Francisella novicida-*infected BMDMs are not due to differences in intracellular bacterial replication

After observing that IL-1β levels were much lower following infection with *F*. *tularensis* LVS than with *F*. *novicida,* we investigated whether this discrepancy was due to differences in intracellular bacterial replication. To determine this, wild-type and *Aim2^−/−^* BMDMs, and immortalized wild-type as well as *Gsdmd^−/−^* BMDMs were infected with *F*. *tularensis* LVS or *F*. *novicida* at 100 MOI. The BMDMs were lysed at 6- and 24-h post-infection to enumerate intracellularly replicated bacteria. It was found that equal numbers of *F*. *tularensis* LVS and *F*. *novicida* were recovered from infected wild-type, *Aim2^−/−^* and *Gsdmd^−/−^* macrophages at 6- and 24-h post-infection (Figure 1C). These results demonstrate that the differences in IL-1β levels in *F*. *tularensis* LVS- and *F*. *novicida-*infected BMDMs are not due to differences in the numbers of intracellular bacteria.

### *Francisella tularensis-*infected BMDMs exhibit lower cell death than *Francisella novicida-*infected BMDMs

Previous studies have shown that *F*. *novicida* infection induces pyroptotic cell death, as indicated by elevated LDH release in the culture supernatants (Fernandes-Alnemri et al., 2010; Man et al., 2015, 2016). To investigate this further, we measured LDH release as a readout for pyroptosis in wild-type, *Aim2^−/−^* and immortalized *Gsdmd^−/−^* BMDMs infected with *F*. *tularensis* LVS or *F*. *novicida* at 24 h post-infection. In wild-type BMDMs, nearly 40% of *F*. *tularensis* LVS*-*infected BMDMs underwent cell death, as compared to almost 70% of *F*. *novicida-*infected BMDMs. Additionally, unlike *F*. *tularensis* LVS-infected BMDMs, the cell death of *F*. *novicida-*infected BMDMs was dependent on Aim2. Furthermore, the cell death observed in *F*. *novicida* was independent of GsdmD, as the extent of cell death in the immortalized *Gsdmd^−/−^* BMDMs was similar to that observed for wild-type BMDMs infected with *F*. *novicida* (Figure 1D). Overall, these results indicate that significantly higher *F*. *novicida-*infected BMDMs undergo Aim2-dependent but GsdmD-independent cell death than *F*. *tularensis* LVS-infected BMDMs.

### *Francisella tularensis* LVS-infected BMDMs produce low levels of IFN-β

**Figure 2:**
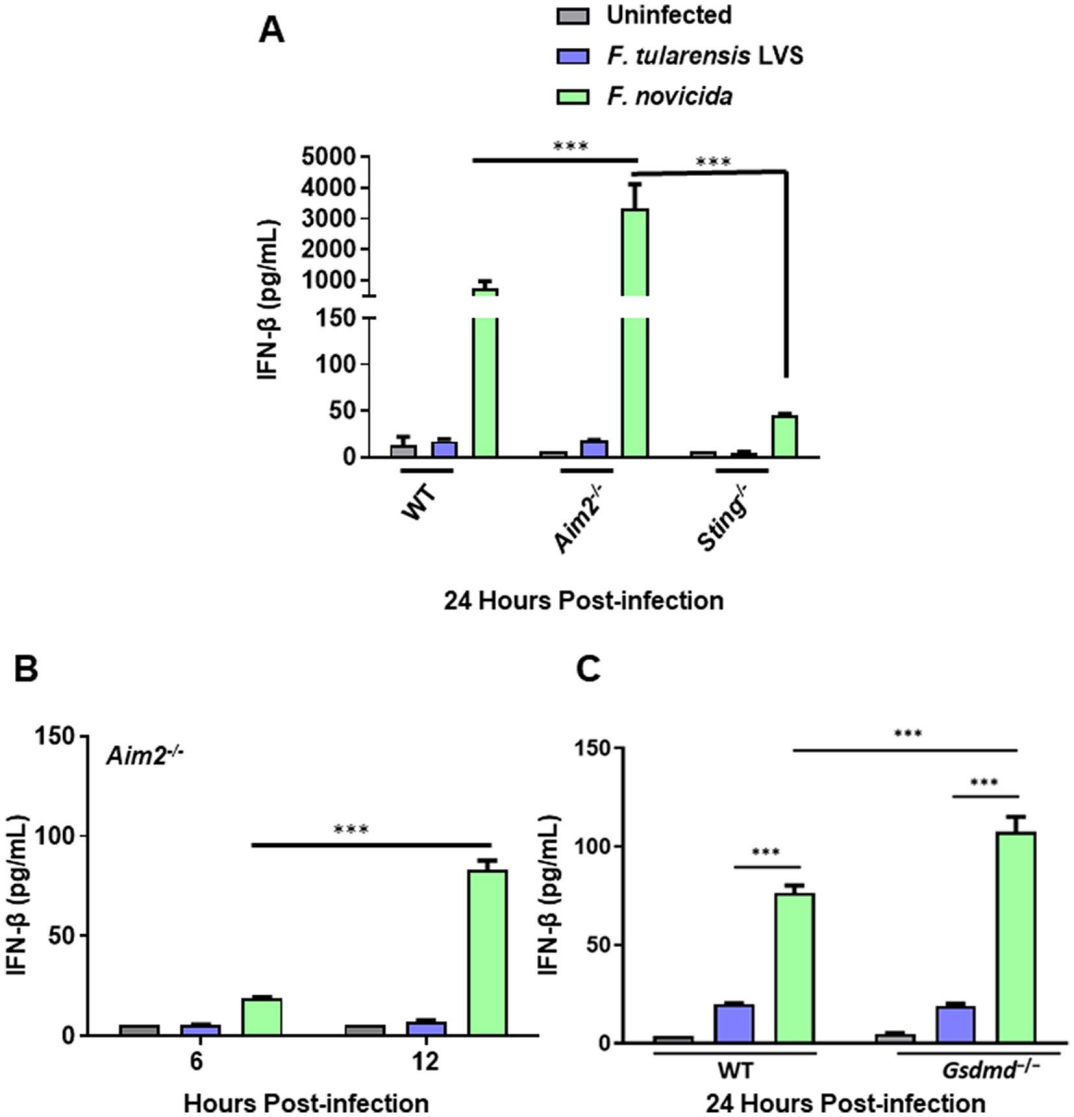
*Francisella tularensis* LVS-infected macrophages produce low levels of IFN-β. Wild-type (WT), *Aim2^−/−^* and *Sting^−/−^* BMDMs **(A,B)** and immortalized WT and *Gsdmd^−/−^* BMDMs **(C)** were infected with *F*. *tularensis* LVS, or *F*. *novicida* (MOI 100). The culture supernatants were analyzed at 6 and 12 h **(B)** and 24 h post-infection **(A,C)** for secreted levels of IFN-β by ELISA. Results are expressed as pg./mL (mean ± SEM) and are cumulative from three independent experiments with up to six biological replicates. The *p* values were determined using one-way ANOVA. ^***^p < 0.001.

Our previous results demonstrated that *F*. *tularensis* LVS weakly activates Aim2 inflammasome, which may impair the downstream events such as cleavage of GsdmD, pyroptotic cell death, and the release of the secreted active form of IL-1β. We next examined the impact of *F*. *tularensis* LVS infection on the cGAS-STING pathway upstream of Aim2 by determining the levels of IFN-β in wild-type, *Aim2^−/−^, Sting^−/−^* and immortalized *Gsdmd*^−/−^ BMDMs. The results show that similar to IL-1β, *F*. *tularensis* LVS induces small quantities of IFN-β in the wild-type BMDMs, and these levels were similar to those observed in *Aim2^−/−^* BMDMs. However, no detectable levels of IFN-β were observed in *Sting^−/−^* BMDMs infected with *F*. *tularensis* LVS. In contrast, *F*. *novicida-*infected wild-type BMDMs produced very high levels of IFN-β in a STING-dependent manner, and these levels remained significantly elevated in *Aim2^−/−^* BMDMs at 12 and 24 post-infection (Figures 2A,B) and in immortalized *Gsdmd^−/−^* BMDMs 24 h post-infection (Figure 2C). Overall, these results demonstrate that BMDMs infected with *F*. *tularensis* LVS produce very low levels of IFN-β compared to *F*. *novicida-*infected cells in a STING-dependent manner. These results suggest that suppression or low activation of the Aim2-inflammasome in *F*. *tularensis* LVS-infected BMDMs could result from a weak activation of the cGAS-STING pathway.

### The introduction of exogenous *Francisella tularensis* LVS DNA in *Francisella tularensis* LVS-infected BMDMs does not lead to enhanced IL-1β levels

**Figure 3:**
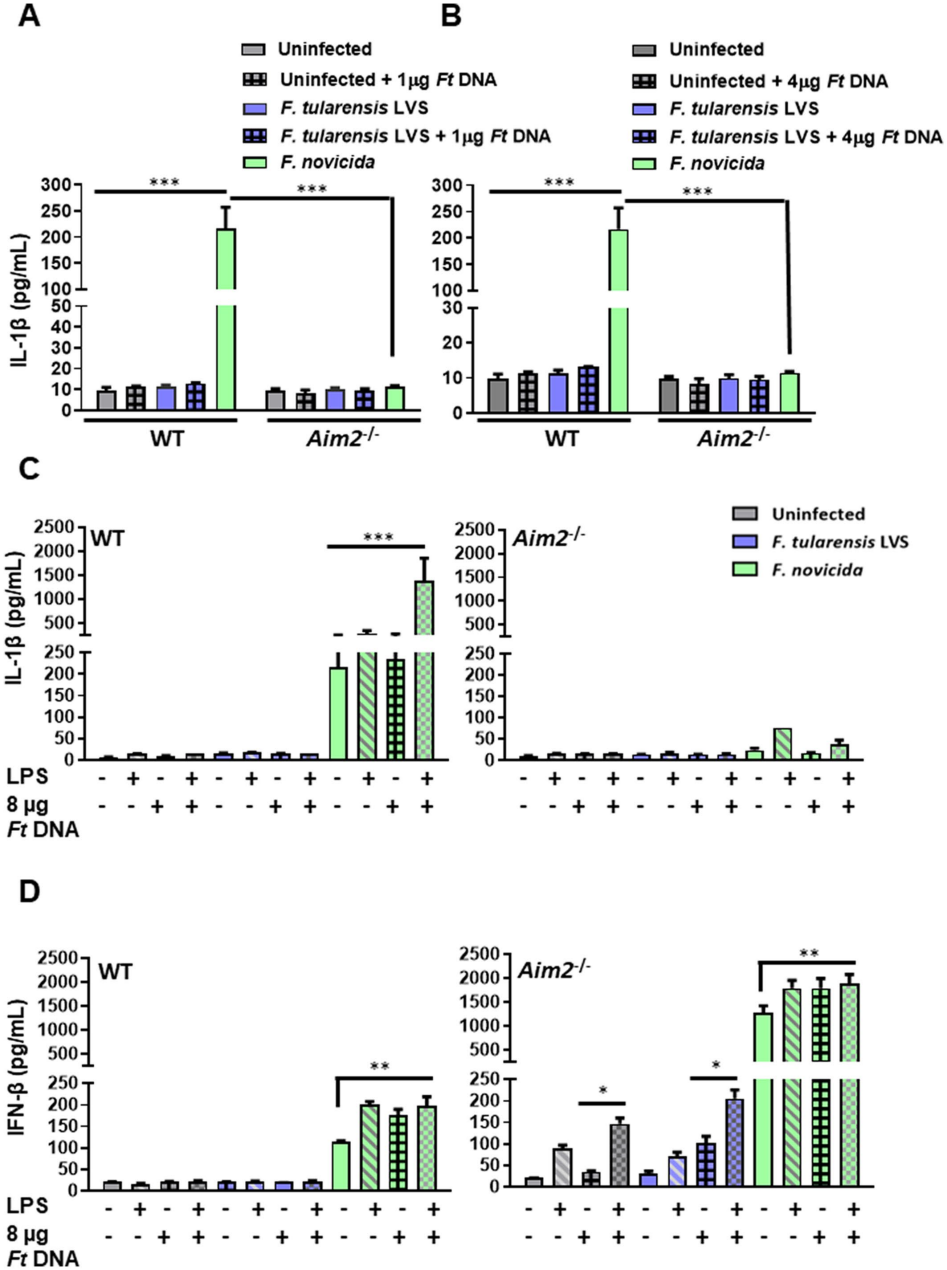
The introduction of exogenous *Francisella tularensis* LVS DNA and/or stimulation with a potent TLR4 agonist does not lead to enhanced IL-1β levels in *F*. *tularensis* LVS-infected macrophages. Wild-type (WT) and *Aim2^−/−^* BMDMs were infected (MOI 100) with *F*. *tularensis* LVS or *Francisella novicida* for 2 h, then transfected with **(A)** 1 μg and **(B)** 4 μg of *F*. *tularensis* LVS DNA. The culture supernatants from these cells were analyzed 24 h post-infection for the levels of secreted IL-1β by ELISA. Results are expressed as pg./mL (mean ± SEM) and are cumulative of two independent experiments with a total of up to six biological replicates. The *p* values were determined using one-way ANOVA. ^***^p < 0.001. LPS-primed or unprimed WT and *Aim2^−/−^* BMDMs were infected with *F*. *tularensis* LVS or *F*. *novicida* (MOI 100) for 2 h, then transfected with 8 μg/mL of *F*. *tularensis* LVS DNA. Culture supernatants were analyzed for the levels of secreted IL-1β **(C)** and IFN-β **(D)** at 24 h post-infection by ELISA. Results are expressed as pg/mL (mean ± SEM) and are cumulative of two independent experiments with up to six replicates. The *p* values were determined using one-way ANOVA. ^*^p < 0.05; ^**^p < 0.01; ^***^p < 0.001.

DNA isolated from *F*. *tularensis* LVS (1 or 4 μg; 1 μg DNA = ~ 1 × 10^6^ genomic copies of *F*. *tularensis* LVS) was introduced into the wild-type and *Aim2^−/−^* BMDMs infected with *F*. *tularensis* LVS. *Francisella novicida* infected, uninfected, and non-DNA transfected BMDMs were used as additional controls. The activation of Aim2 inflammasome was determined by measuring the secretion of IL-1β in the culture supernatants of these BMDMs. The results showed that both wild-type and *Aim2^−/−^* BMDMs either untransfected or transfected with *F*. *tularensis* LVS DNA and infected with *F*. *tularensis* LVS produced similarly low levels of IL-1β as compared to untransfected and *F*. *novicida-*infected BMDMs. As observed earlier, the production of IL-1β in *F*. *novicida-*infected BMDMs was Aim2-dependent (Figures 3A,B).

We conducted further experiments with a higher DNA concentration (8 μg) and with or without prior priming with lipopolysaccharide (LPS) derived from *Escherichia coli,* which is a potent TLR4 agonist. As an additional control, *F*. *tularensis* LVS DNA was also added to *F*. *novicida-*infected cells. The culture supernatants were then analyzed for the secreted levels of IL-1β at 24 h post-infection. The results showed that introducing 8 μg of *F*. *tularensis* LVS DNA with or without the LPS priming did not affect the levels of secreted IL-1β in uninfected and *F*. *tularensis* LVS-infected wild-type or the *Aim2^−/−^* BMDMs. Moreover, only the introduction of *F*. *tularensis* LVS DNA into LPS-primed *F*. *novicida-*infected BMDMs promoted robust inflammasome activation as indicated by higher IL-1β secretion as compared to the untransfected *F*. *novicida* controls (217.047 ± 40.3 vs. 1392.64 ± 969 pg./mL, respectively). This significant increase in IL-1β levels also confirmed that DNA transfection was successful, which was enhanced further upon priming the BMDMs with *E*. *coli* LPS in *F*. *novicida-*infected BMDMs (Figure 3C). These results demonstrate that suppression or weak activation of the Aim2-inflammasome by *F*. *tularensis* LVS was not relieved even after the introduction of exogenous *F*. *tularensis* LVS DNA or by LPS priming.

### The addition of exogenous *Francisella tularensis* LVS DNA leads to enhanced IFN-β levels only in *Aim2*^−/−^ BMDMs

To examine whether the introduction of *F*. *tularensis* LVS DNA results in cGAS-STING-dependent production of IFN-β, we determined the levels of secreted IFN-β. The results showed that the levels of IFN-β in untransfected or LPS-primed wild-type BMDMs, infected with *F*. *tularensis* LVS and treated with *F*. *tularensis* LVS DNA, were similar to those observed for the uninfected controls (Figure 3D). However, the LPS-primed, DNA-treated, or LPS-primed and DNA-treated, and *F*. *novicida-*infected BMDMs all induced significantly higher levels of IFN-β as compared to the untreated *F*. *novicida-*infected BMDMs. These results indicated that *F*. *tularensis* LVS possesses suppressive mechanisms, which, even after *E*. *coli* LPS priming or the introduction of *F*. *tularensis* LVS DNA, do not result in the production of IFN-β except in the *Aim2^−/−^* BMDMs.

We next examined the impact of IFN-β production in untreated, LPS-primed, *F*. *tularensis* LVS DNA-treated, and *F*. *tularensis* LVS or *F*. *novicida* infected *Aim2^−/−^* BMDMs. It was observed that LPS priming and introduction of *F*. *tularensis* LVS DNA in uninfected *Aim2^−/−^* BMDMs induced significantly higher levels of IFN-β as compared to untreated, LPS-primed or DNA-treated alone counterparts. A similar trend with significantly higher IFN-β levels were observed in LPS-primed, DNA-treated, and *F*. *tularensis* LVS or the *F*. *novicida-*infected *Aim2^−/−^* BMDMs (Figure 3D). These results indicate *F*. *tularensis* LVS-infected *Aim2^−/−^* BMDMs do respond to LPS priming and introduction of *F*. *tularensis* LVS DNA. However, these responses are muted in the wild-type BMDMs.

### Stimulation with *Escherichia coli* LPS and/or the addition of exogenous *Francisella tularensis* LVS DNA does not lead to enhanced TNF-α response in *Francisella tularensis* LVS-infected BMDMs

**Figure 4:**
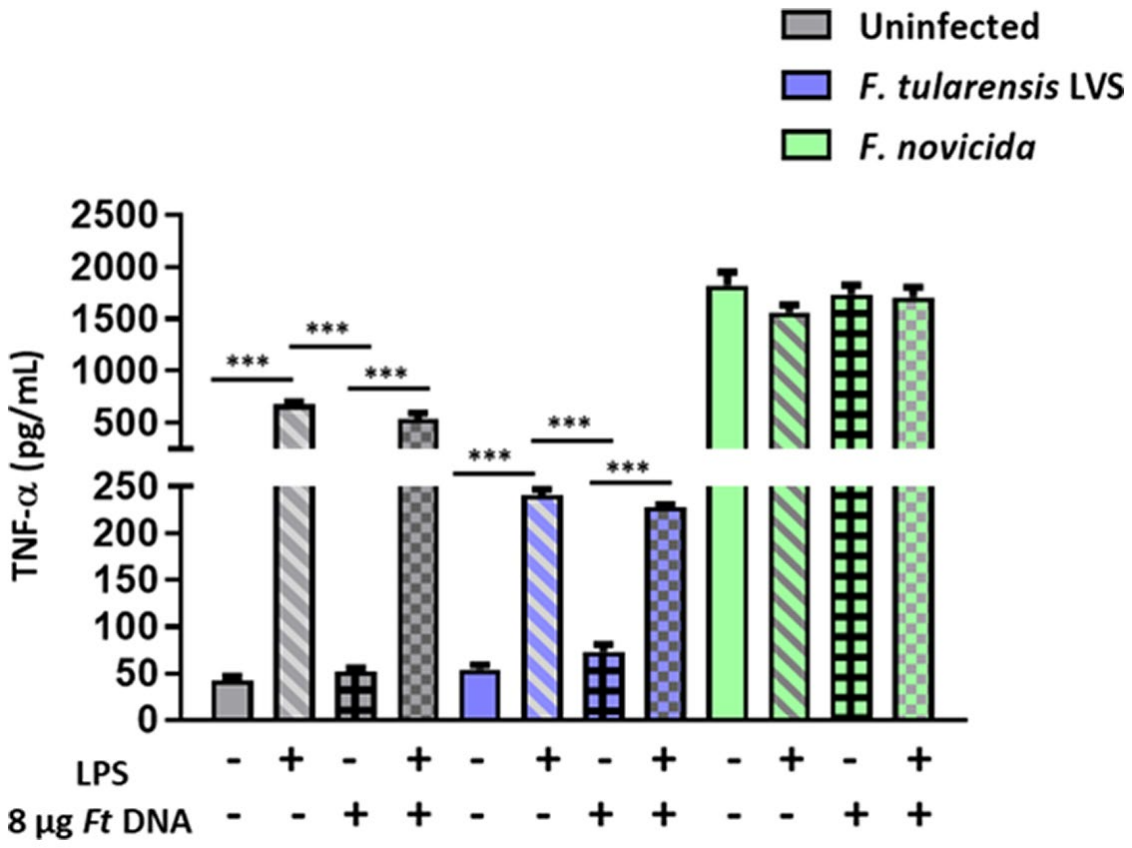
Stimulation with *Escherichia coli* LPS and/or the introduction of exogenous *Francisella tularensis* LVS DNA does not lead to enhanced TNF-α response in *F*. *tularensis* LVS-infected BMDMs. *Escherichia coli* LPS-primed or unprimed wild-type (WT) BMDMs were infected with *F*. *tularensis* LVS, or *F*. *novicida* at (MOI 100) for 2 h, then transfected with 8 μg/mL of *F*. *tularensis* LVS (*Ft*) DNA. Culture supernatants were analyzed for the levels of TNF-α at 24 h post-infection by ELISA. Results are expressed as pg./mL (mean ± SEM) and are cumulative from two independent experiments with up to six biological replicates. The *p* values were determined using one-way ANOVA. ^***^p < 0.001.

We next determined if *F*. *tularensis* LVS exerts its suppressive effect only on cGAS-STING-dependent Aim2-inflammasome signaling or if other signaling pathways are similarly impacted. We specifically looked at the levels of TNF-α, a readout for the activation of the NF-κB signaling pathway. Very high levels of TNF-α were observed in BMDMs primed with *E*. *coli* LPS compared to the uninfected or BMDMs transfected with *F*. *tularensis* LVS DNA. The TNF-α levels did not increase when the BMDMs were treated with both *E*. *coli* LPS plus *F*. *tularensis* LVS DNA and infected with *F*. *tularensis* LVS. Surprisingly, the levels of TNF-α in LPS primed alone or those treated with both LPS and DNA and infected with *F*. *tularensis* LVS were significantly lower than those observed for their respective uninfected controls. Significantly high levels of TNF-α were observed in all groups of *F*. *novicida-*infected BMDMs than those observed for uninfected or *F*. *tularensis* LVS-infected BMDMs. Also, more or less similar levels were observed in *F*. *novicida-*infected BMDMs, irrespective of the treatment (Figure 4). Together, these results again highlight the differences between *F*. *tularensis* LVS and *F*. *novicida* in the type of innate immune response induced by the infected macrophages. Additionally, these results also confirm that *F*. *tularensis* is capable of suppressing an ongoing proinflammatory immune response.

### Mitophagy is induced upon *Francisella tularensis* LVS infection

**Figure 5:**
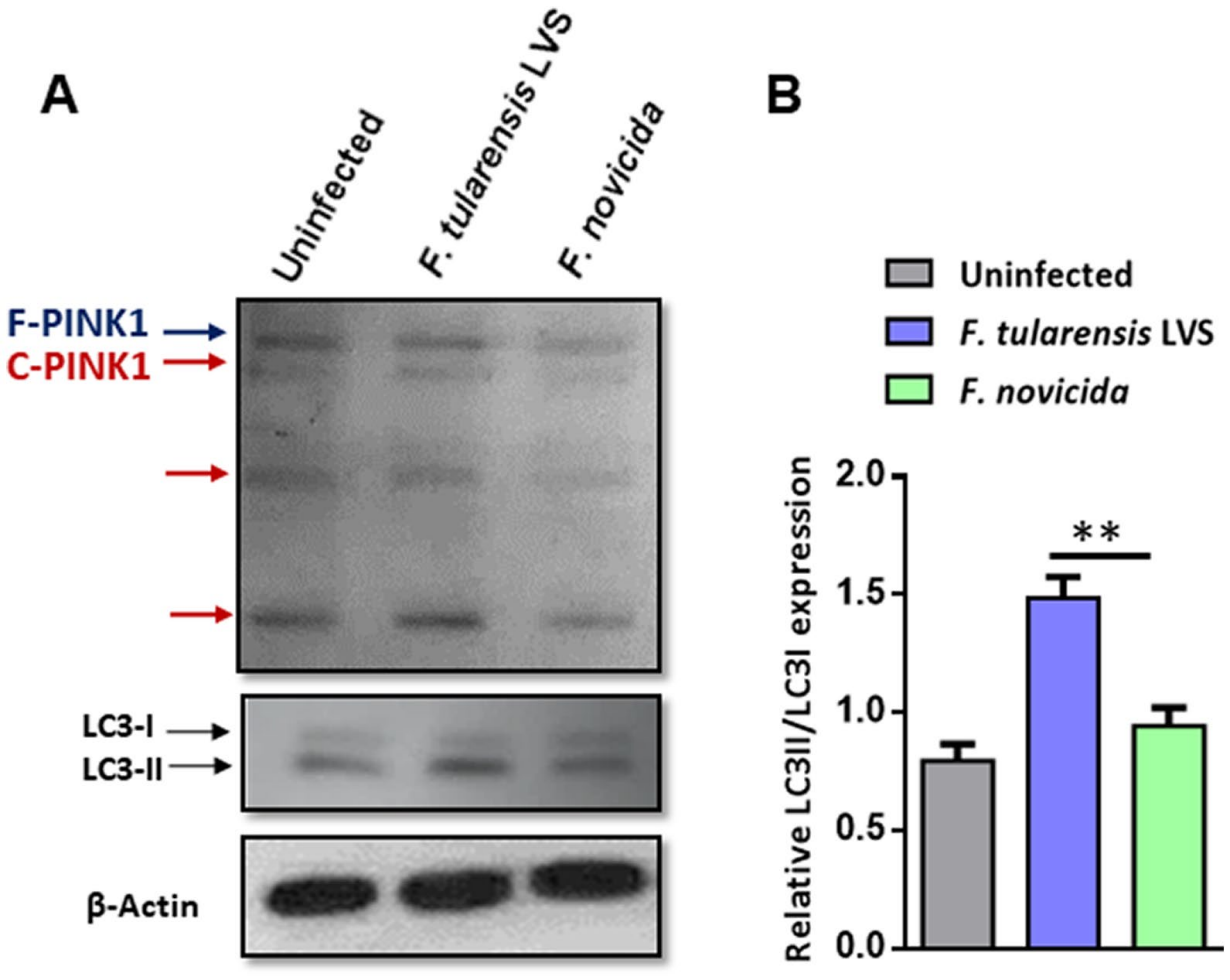
Mitophagy is induced upon *Francisella tularensis* LVS infection. **(A)** Western blot analysis of uninfected and BMDMs infected with *F*. *tularensis* LVS or *Francisella novicida* (MOI 100). Lysates were collected 6 h post-infection and subjected to SDS-PAGE, then blotted for PINK1, LC3 I/II, and β-Actin. F-PINK1, full-length PINK1, and C-PINK1, cleaved PINK1. A representative blot out of three is shown. **(B)** Relative LC3II/LC3I expression was quantitated from three blots. The *p* values were determined using one-way ANOVA. ^**^p < 0.01.

Mitophagy is a process in which the damaged mitochondria are selectively removed by autophagy. We investigated the levels of PINK1 protein and LC3A/B in wild-type BMDMs infected with *F*. *tularensis* LVS or *F*. *novicida* (MOI 100) 6 h post-infection by western blot analysis. The western blot results showed higher levels of full-length and cleaved PINK1 in *F*. *tularensis* LVS-infected BMDMs compared to uninfected or *F*. *novicida-*infected BMDMs (Figure 5A). This increase was associated with an increase in LC3-II to LC3-I ratio in *F*. *tularensis* LVS-infected macrophages compared to the uninfected control or the *F*. *novicida-* infected cells (Figure 5B). These results suggest that PINK1-mediated autophagy may be involved in removing stressed/damaged mitochondria during *F*. *tularensis* LVS infection.

**Figure 6:**
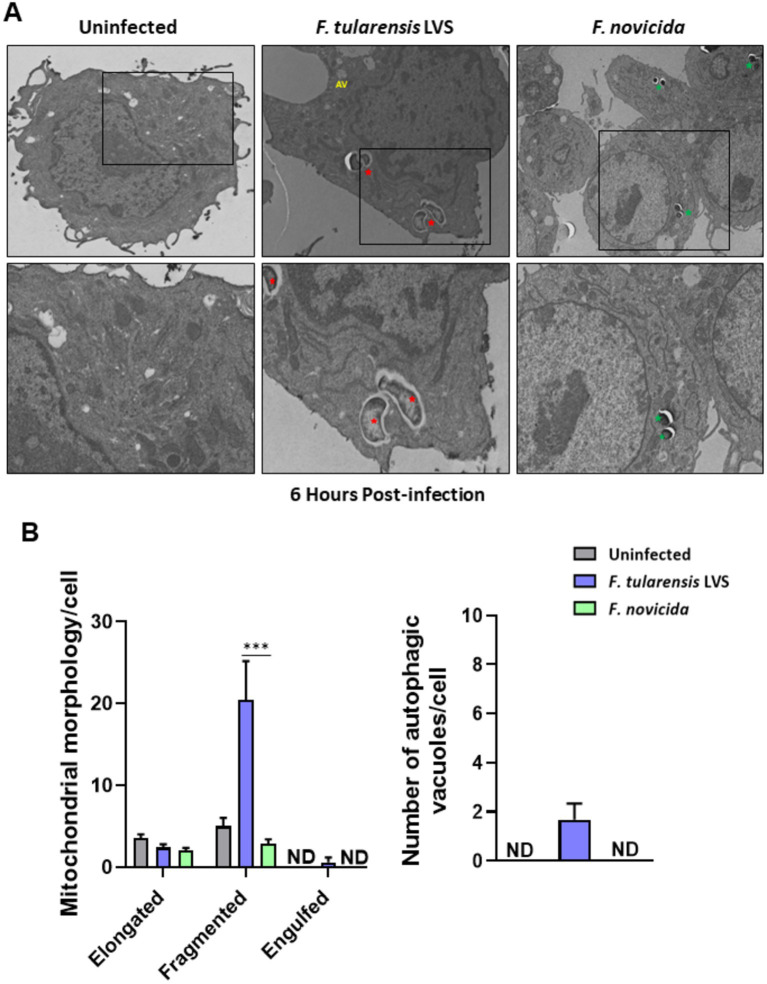
Increased mitophagy is observed in BMDMs infected with *Francisella tularensis* LVS 6 h post-infection. **(A)** Representative transmission electron microscopy images randomly selected from uninfected, *F*. *tularensis* LVS, and *Francisella novicida* infected BMDMs 6 h post-infection are shown. The identification of *F*. *tularensis* LVS and *F*. *novicida* was done on the basis of the size, shape, and location of the bacteria within macrophages. *Francisella tularensis* is represented by red stars and *F*. *novicida* is represented by green stars. The black boxes indicate the higher magnification of the areas shown in lower panels. **(B)** Quantitation of mitochondrial morphology and autophagic vacuoles per cell. Representative results from three independent experiments are shown. The *p* values were determined using one-way ANOVA. ^***^p < 0.001. ND, not detected; AV, autophagic vacuoles.

**Figure 7:**
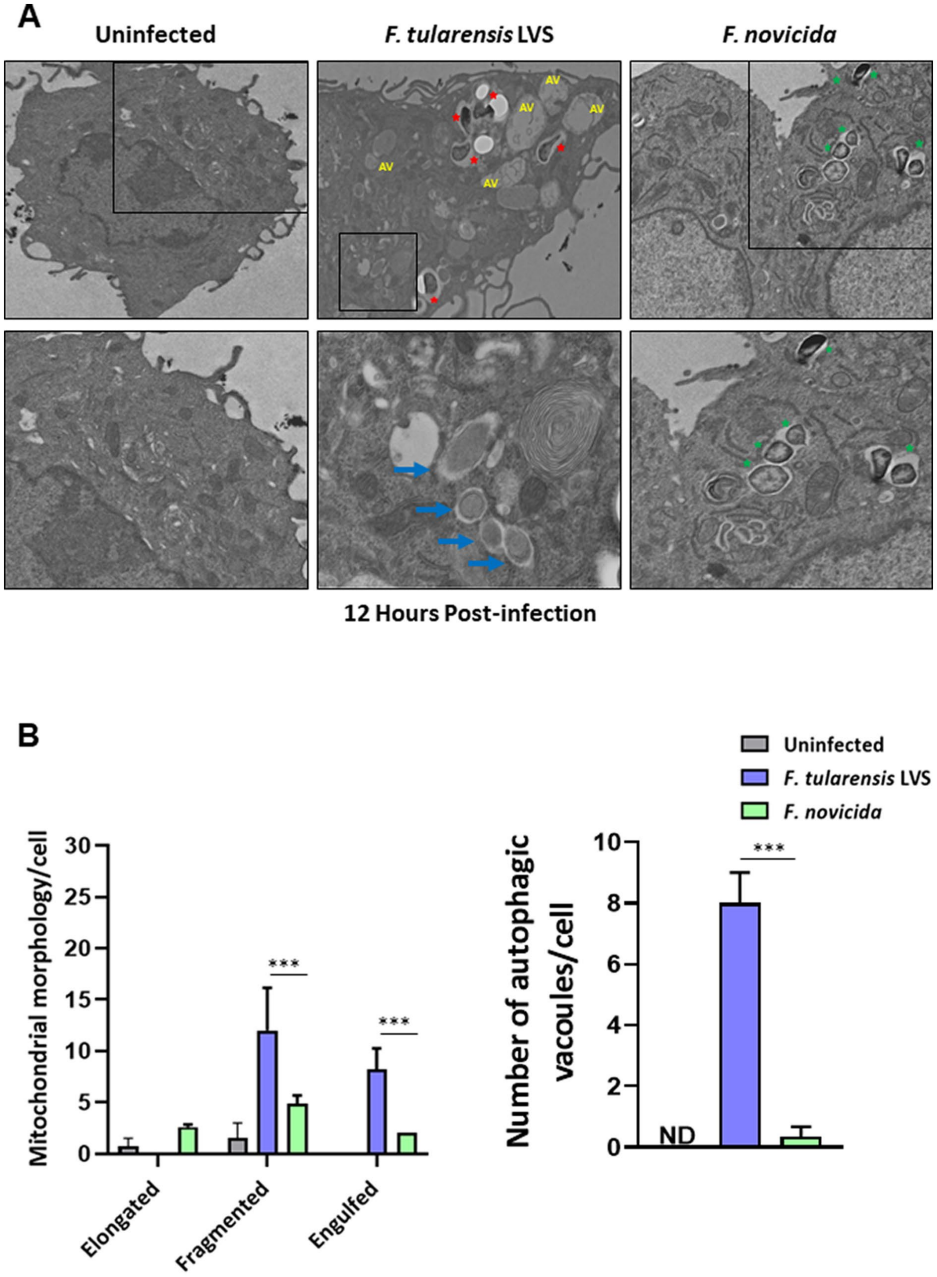
Increased mitophagy is observed in BMDMs infected with *Francisella tularensis* LVS 12 h post-infection. **(A)** Representative transmission electron microscopy images randomly selected from uninfected, *F*. *tularensis* LVS, and *F*. *novicida* infected BMDMs 12 h post-infection are shown. *Francisella tularensis* is represented by red stars and *F*. *novicida* is represented by green stars. Autophagic vacuoles are indicated by blue arrows. The black boxes indicate the higher magnification of the areas shown in lower panels. **(B)** Quantitation of mitochondrial morphology and autophagic vacuoles per cell. Representative results from three independent experiments are shown. The *p* values were determined using one-way ANOVA. ^***^p < 0.001. ND, not detected; AV, autophagic vacuoles.

We investigated further by examining BMDMs infected with *F*. *tularensis* LVS or *F*. *novicida* at 6- and 12-h post-infection for the signs of mitochondrial damage or the induction of mitophagy by TEM. Assessment of mitochondrial morphology in the uninfected macrophages revealed tubular networks, while *F*. *tularensis* LVS-infected cells possessed fragmented and round mitochondria as early as one-hour post-infection (not shown). Also, *F*. *tularensis* LVS-infected macrophages showed an increase in autophagic vacuoles, some of which contained the engulfed mitochondria. Conversely, macrophages infected with *F*. *novicida* showed mostly tubular and some round, swollen mitochondria, but no engulfed mitochondria were observed (not shown). At 6 h post-infection, mitochondrial fragmentation increased significantly in *F*. *tularensis* LVS-infected cells. Low levels of mitochondrial fragmentation were also observed in both uninfected and *F*. *novicida*-infected BMDMs. Furthermore, *F*. *tularensis* LVS infection was associated with the presence of higher numbers of autophagic vacuoles, while none were detected in uninfected and *F*. *novicida*-infected BMDMs (Figures 6A,B). At 12 h post-infection, the number of fragmented mitochondria per cell decreased from that observed at 6 h, and this was associated with higher numbers of autophagic vacuoles containing damaged mitochondria and autophagosomes in *F*. *tularensis* LVS compared to the *F*. *novicida* infected BMDMs. Additionally, the numbers of autophagosomes were significantly higher in *F*. *tularensis* LVS-infected BMDMs at 12 h post-infection than those observed at 6 h (Figures 7A,B). These results indicate that enhanced mitophagy is induced during *F*. *tularensis* LVS-infected than *F*. *novicida-*infected macrophages.

## Discussion

The mechanisms and signaling pathways leading to the activation of Aim2 inflammasome have been elegantly elucidated using the *F*. *novicida* strain as a model. However, these mechanisms remain unknown for *F*. *tularensis* strains. This study aimed to understand the mechanisms of suppression of Aim2 inflammasome by *F*. *tularensis* LVS and to investigate if the mechanisms known for *F*. *novicida* are also operative in *F*. *tularensis* strains.

Our results showed that in *F*. *tularensis* LVS-infected BMDMs, very low levels of IL-1β were produced in an Aim2-dependent manner than in *F*. *novicida*-infected BMDMs. The differences in IL-1β levels in *F*. *tularensis* LVS and *F*. *novicida-*infected BMDMs were not due to differences in their growth kinetics since both strains grew similarly in wild-type BMDMs or BMDMs lacking inflammasome sensors. However, there were differences in the extent of cell death, as indicated by the LDH release in macrophages infected with *F*. *tularensis* LVS and *F*. *novicida*. In an *in vivo* study (Hall et al., 2008) comparing the pathogenesis of *F*. *tularensis* LVS and *F*. *novicida* side-by-side, it was reported that there are differences in the propensity of these two *Francisella* subspecies to infect cells. The number of cells infected by *F*. *novicida* at the initiation of infection is higher than those infected by *F*. *tularensis* LVS. Furthermore, no progressive increase in the number of *F*. *novicida*-infected cells is observed. On the other hand, despite the number of cells initially infected by *F*. *tularensis* LVS are low, an increasing number of cells continue to become infected as the infection progresses. Therefore, the number of both *F*. *novicida* and *F*. *tularensis* observed during the later stages of infection are identical. Moreover, *F*. *novicida* causes a more rapid killing of the cells than *F*. *tularensis* LVS, as observed by the rapid depletion of macrophages and dendritic cells. Our *in vitro* results mirror this *in vivo* profile and show recovery of identical numbers of both *F*. *tularensis* LVS and *F*. *novicida* from the infected macrophages, however, they reveal significant differences in cell death between *F*. *tularensis* and *F*. *novicida*-infected macrophages.

Gasdermin D plays a crucial role in pyroptosis, which is characterized by the formation of pores in the cell membrane. In addition to IL-1β and IL-18, these pores facilitate the rapid release of intracellular contents, including the lytic cell death marker, LDH, into the extracellular environment (Banerjee et al., 2018). Interestingly, in *Gsdmd*^−/−^ macrophages infected with *F*. *novicida,* the levels of LDH were similar to those observed in wild-type macrophages (Figure 1D). These results corroborate a previous study that has shown that *Gsdmd*-deficient macrophages, similar to wild-type macrophages, exhibit increased LDH release despite their inability to undergo GsdmD-dependent pyroptosis. This robust GsdmD-independent LDH release has been reported to be due to cell lysis caused by necrotic rather than pyroptotic cell death in *Gsdmd*^−/−^ macrophages (Schneider et al., 2017).

Previous studies have shown that IFN-β production by activated cGAS-STING pathway is a prerequisite for optimal Aim2 inflammasome activation in *F*. *novicida-*infected macrophages (Henry et al., 2007; Storek et al., 2015), and that IFN-β production is negatively regulated via Aim2 inflammasome (Corrales et al., 2016; Nakaya et al., 2017; Banerjee et al., 2018; Yan et al., 2018). Our results revealed significantly lower levels of IFN-β in *F*. *tularensis* LVS-infected BMDMs than *F*. *novicida-*infected BMDMs in a STING-dependent manner, suggesting that suppression/weak activation of cGAS-STING pathway by *F*. *tularensis* LVS may result in suppression/weak activation of the Aim2 inflammasome. However, the levels of IFN-β in *F*. *tularensis* LVS-infected BMDMs did not change irrespective of the presence or absence of Aim2 or GsdmD and remained significantly lower than those observed for *F*. *novicida-*infected BMDMs. On the other hand, higher levels of IFN-β in *Aim2^−/−^* and *Gsdmd^−/−^* BMDMs infected with *F*. *novicida* were observed as compared to their wild-type counterparts. These results indicated that even trace amounts of DNA that can be sensed by the c-GAS-STING pathway might not be released during the replication of *F*. *tularensis,* which may serve as a potential mechanism to prevent activation of the Aim2 inflammasome. Furthermore, these results also indicate potential differences that could exist in the ability of *F*. *novicida* and *F*. *tularensis* LVS DNA to stimulate or be recognized by the cGAS-STING pathway. Previous studies have shown that AT-rich motifs in the genome of *Plasmodium falciparum* are sensed by the STING pathway, leading to a potent type-1 interferon response (Sharma et al., 2011). It would be of great interest to investigate if the existence of similar AT-rich motifs in higher abundance in *F*. *novicida* DNA than in *F*. *tularensis* LVS DNA contributes to differential activation of cGAS-STING pathway resulting in higher levels of IFN-β.

We next sought to elucidate the mechanisms behind suppression/weak activation of the Aim2 inflammasome in *F*. *tularensis* LVS-infected BMDMs. We hypothesized that the low levels of IFN-β and IL-1β observed upon *F*. *tularensis* LVS infection were due to insufficient release of bacterial DNA. To investigate this, we introduced exogenous *F*. *tularensis* DNA into the cytosol of BMDMs infected with *F*. *tularensis* to activate the cGAS-STING signaling pathway and prime the Aim2 inflammasome for IFN-β and IL-1β, respectively. We anticipated that this approach would bring the levels of IL-1β in *F*. *tularensis* LVS-infected macrophages similar to those observed for *F*. *novicida-*infected counterparts. However, even with the introduction of exogenous *F*. *tularensis* LVS DNA, the levels of secreted IL-1β remained similar to untransfected and *F*. *tularensis* LVS-infected BMDMs, indicating that additional exogenous DNA failed to activate Aim2-dependent IL-1β production in *F*. *tularensis* LVS-infected BMDMs. These intriguing results prompted us to investigate further why exogenous DNA did not activate the Aim2-inflammasome in *F*. *tularensis* LVS-infected BMDMs.

Activation of the inflammasome is a complex process that requires two signals. Signal-one or the priming signal is provided by a TLR ligand that activates the NF-κB pathway for the transcriptional upregulation of certain inflammasome components, including *pro-IL-1β*. Signal-two, or the activation signal, is provided by microbial components, endogenous molecules, and/or stress signals that are recognized by the inflammasome sensors such as Aim2 or Nlrp3 to activate the inflammasome (Broz and Dixit, 2016). Since *F*. *tularensis* also suppress TLR-dependent signaling pathways (Dotson et al., 2013), we reasoned that low levels of IL-1β in *F*. *tularensis* LVS-infected BMDMs could be due to a lack of signal-one or that the amount of introduced DNA is not sufficient for the activation of Aim2 as signal-two. We next primed the cells by treating them with a potent TLR4 ligand, *E*. *coli* LPS, and introduced a higher DNA concentration (8 μg) of exogenous *F*. *tularensis* LVS DNA. However, neither priming with *E*. *coli* LPS nor a higher quantity of exogenous *F*. *tularensis* LVS DNA improved the levels of IL-1β. Contradictorily, providing both signal-one and -two further elevated the levels of IL-1β in *F*. *novicida-*infected BMDMs in an Aim2-dependent fashion. Overall, these results demonstrate that suppression/weak activation of Aim2 inflammasome by *F*. *tularensis* LVS was not relieved even after the introduction of exogenous *F*. *tularensis* LVS DNA or boosting the signal-one by LPS priming. However, LPS priming resulted in the production of TNF-α in *F*. *tularensis* LVS-infected BMDMs at lower levels compared to the uninfected cells, while TNF-α levels were produced at extremely high levels during *F*. *novicida* infection irrespective of LPS stimulation. These results confirm previous findings about the ability of *F*. *tularensis* to suppress activated macrophages (Dueñas et al., 2006; Gillette et al., 2014).

We further investigated the mechanism responsible for suppression/weak activation of Aim2 inflammasome that remained elusive from the preceding studies. We hypothesized that DNA coming from some other source might also contribute to the higher levels of inflammasome-dependent cytokines in *F*. *novicida-*infected BMDMs and that *F*. *tularensis* may have evolved mechanisms to cause immunosuppression. This notion was investigated next.

Studies have shown that during stress, there is a loss of membrane potential, increased release of mitochondrial DNA (mtDNA), decreased production of ATP, and increased fragmentation of dysfunctional mitochondria (Murphy, 2009; Larsen et al., 2012; Youle and Van Der Bliek, 2012; Friedman and Nunnari, 2014; Garza-Lombó et al., 2020). A previous study has demonstrated the ability of *F*. *tularensis* SchuS4 to manipulate mitochondrial activity early during infection, enhancing mitochondrial functions by impairing the metabolic shift from oxidative phosphorylation to glycolysis (Jessop et al., 2018). *Francisella tularensis* LVS is also capable of maintaining mitochondrial integrity during neutrophil infection *in vivo* to delay apoptosis (McCracken et al., 2016). Moreover, mtDNA was not detected in isolated mice leukocytes 2 days post-infection with *F*. *tularensis* LVS but started to appear after the third day of infection, reaching significantly higher levels on day six post-infection (Singh et al., 2017). The appearance of mtDNA is associated with an increase in proinflammatory cytokine response. Additionally, the enhanced inflammasome activation during *F*. *novicida* infection *in vitro* is found to be mediated by mitochondrial reactive oxygen species (mROS), suggesting the accumulation of stressed mitochondria (Crane et al., 2014). Collectively, these studies demonstrated that mtDNA released from stressed mitochondria might play a key role in activating the innate immune responses during *Francisella* infection. Based on these observations, we postulated that dysfunctional mitochondria might be removed early during *F*. *tularensis* LVS infection to avoid stimulating Aim2-mediated responses by the leaked mtDNA.

Our results indicate that mitophagy is induced during *F*. *tularensis* LVS, but not *F*. *novicida* infection. The results suggest a role for the PINK1/Parkin pathway, which converts LC3-I to LC3-II to induce mitophagy in *F*. *tularensis* LVS-infected macrophages. Furthermore, our electron microscopy results indicate mitochondrial fragmentation and mitophagy in *F*. *tularensis* LVS-infected BMDMs compared to their *F*. *novicida*-infected counterparts. However, a detailed investigation is warranted to understand the pathways and mechanisms involved in the induction of mitophagy and its direct association with the suppression of the Aim2 inflammasome in *F*. *tularensis* LVS-infected macrophages.

However, the question remains: why is there enhanced mitophagy in *F*. *tularensis*-infected BMDMs compared to *F*. *novicida-*infected ones? One potential explanation could be that several TCA cycle components are missing in *F*. *tularensis,* and these strains rely extensively on the host cell metabolism, which stresses out the mitochondria. *Francisella novicida* possesses all TCA cycle components and may impose less stress on mitochondria (Rohmer et al., 2007; Gesbert et al., 2015; Ziveri et al., 2017). Another question is why dysfunctional mitochondria are removed from *F*. *tularensis* LVS-infected BMDMs but not from those infected with *F*. *novicida*. M1 macrophages and activated DCs have an incomplete TCA cycle, and a pathogen with a deficiency in nine amino acids biosynthetic pathways may induce additional stress on mitochondria and the infected cell, resulting in the induction of autophagic pathways (Dieppedale et al., 2013; Williams and O’Neill, 2018; Breda et al., 2019). However, more detailed work is needed to be done to address these probable explanations.

## Conclusion

This study reveals that *F*. *tularensis* LVS induces low levels of Aim2-dependent IL-1β than those infected with *F*. *novicida*. The suppression/weak activation of the Aim2 inflammasome is due to the suppression of the cGAS-STING DNA-sensing pathway upstream of Aim2 in *F*. *tularensis* LVS-infected BMDMs. The introduction of exogenous *F*. *tularensis* LVS DNA into the cytosol of the infected BMDMs alone or in conjunction with a priming signal provided by a potent TLR4-agonist failed to restore IL-1β levels similar to those observed for *F*. *novicida*. Collectively, the results from this study demonstrate that *F*. *tularensis* suppresses the activation of Aim2 inflammasome, and the mechanisms established for *F*. *novicida* are not operative in *F*. *tularensis* LVS.

## Data availability statement

The original contributions presented in the study are included in the article/Supplementary material, further inquiries can be directed to the corresponding authors.

## Ethics statement

The animal study was reviewed and approved by IACUC New York Medical College.

## Author contributions

MA, ZM, and JM carried out the experiments and data analysis. JY conducted the transmission electron microscopy. MM and CB conceived, coordinated, and supervised the research and wrote the manuscript. All authors contributed to the article and approved the submitted version.

## Funding

This work was supported by National Institutes of Health Grants R56AI101109 and R21AI51277 (CB) and R15AI107698 (MM).

## Conflict of interest

The authors declare that the research was conducted in the absence of any commercial or financial relationships that could be construed as a potential conflict of interest.

## Publisher’s note

All claims expressed in this article are solely those of the authors and do not necessarily represent those of their affiliated organizations, or those of the publisher, the editors and the reviewers. Any product that may be evaluated in this article, or claim that may be made by its manufacturer, is not guaranteed or endorsed by the publisher.
